# Behavioral Modulation by Spontaneous Activity of Dopamine Neurons

**DOI:** 10.3389/fnsys.2017.00088

**Published:** 2017-12-11

**Authors:** Toshiharu Ichinose, Hiromu Tanimoto, Nobuhiro Yamagata

**Affiliations:** ^1^Graduate School of Life Sciences, Tohoku University, Sendai, Japan; ^2^Department of Neuroscience of Disease, Center for Transdisciplinary Research, Niigata University, Niigata, Japan

**Keywords:** spontaneous activity, dopamine, sleep, learning and memory, feeding, sex drive, *Drosophila*

## Abstract

Dopamine modulates a variety of animal behaviors that range from sleep and learning to courtship and aggression. Besides its well-known phasic firing to natural reward, a substantial number of dopamine neurons (DANs) are known to exhibit ongoing intrinsic activity in the absence of an external stimulus. While accumulating evidence points at functional implications for these intrinsic “spontaneous activities” of DANs in cognitive processes, a causal link to behavior and its underlying mechanisms has yet to be elucidated. Recent physiological studies in the model organism *Drosophila melanogaster* have uncovered that DANs in the fly brain are also spontaneously active, and that this activity reflects the behavioral/internal states of the animal. Strikingly, genetic manipulation of basal DAN activity resulted in behavioral alterations in the fly, providing critical evidence that links spontaneous DAN activity to behavioral states. Furthermore, circuit-level analyses have started to reveal cellular and molecular mechanisms that mediate or regulate spontaneous DAN activity. Through reviewing recent findings in different animals with the major focus on flies, we will discuss potential roles of this physiological phenomenon in directing animal behaviors.

## Introduction

Animals need to modify behaviors according not only to the external world but also to their internal states, such as sleep need, hunger, or sexual motivation (Keene et al., 2010; Gorter et al., 2016; Keebaugh et al., 2017). These internal states are encoded in various manners, including ongoing neural activity in the brain. Physiological studies have revealed that these “spontaneously” occurring neural activities often show drastic changes even in the absence of external stimuli (Fox and Raichle, 2007). In this review, we discuss the biological relevance of spontaneous neural activity: how it is regulated and how it modifies behaviors. We define the spontaneous activity as the ongoing neural activity without overt external stimuli, regardless of the properties of the activity, like tonic or burst firing. Activity reflecting self-locomotion is also defined as spontaneous activity in this article.

In mammals, during sleep, electroencephalogram records show characteristic slow waves in the entire cortex (Massimini et al., 2004), which is caused by a spontaneously occurring synchronized neural activity. The slow wave activity is enhanced after sleep deprivation and suppressed after sleep, thereby controlling sleep homeostasis (Tobler and Borbely, 1986; Werth et al., 1996; Finelli et al., 2000; Vyazovskiy et al., 2009). Similar waves also drive rhythmic activity in hippocampus, which is suggested to be critical in memory consolidation (Sirota et al., 2003; Marshall and Born 2007), and a growing number of studies are now revealing how these neural activities occur across various brain regions and how they modify behaviors.

Although many neurotransmitters especially monoamines are reported to control spontaneous activity (Berridge et al., 2012; Dominguez-Lopez et al., 2012; Grace, 2016), we focus on dopaminergic circuits in this review, given converging evidence in identifying spontaneous dopamine signaling as representing states of animals. Dopamine plays a key role in a variety of brain functions such as reward processing, regulation of motivation, or learning and memory (Schultz, 2007). Dopamine functions through both synaptic and volume transmission, thereby enabling it to modulate both intra- and extra- synaptic targets (Rice et al., 2011). Dopamine neurons (DANs) in the midbrain can be characterized by its stimulus-induced phasic firing, the importance of which in behavioral action selection and reward-based learning has been widely acknowledged (Schultz et al., 1997; Tsai et al., 2009; Bromberg-Martin et al., 2010; Cohen et al., 2012; Steinberg et al., 2013; Schultz, 2015). Moreover, a significant number of DANs are known to be spontaneously active (Grace and Bunney, 1984). Studies using microdialysis found that the extracellular dopamine level shows slow fluctuations lasting seconds to minutes without any external stimuli (Schultz, 2007). These dopamine fluctuations are suggested to represent animal states, such as sleep/wake, or motivational state of the animal (Fiorillo et al., 2003; Dahan et al., 2007; Hamid et al., 2016). Dysregulation of dopamine levels causes various mental disorders, suggestive of the crucial role of spontaneously released dopamine in cognition and perception (Krishnan et al., 2007; Cao et al., 2010; Dalley and Roiser, 2012; Chaudhury et al., 2013; Grace, 2016). Although studies in primate and rodent brains have provided us with useful mechanistic insights in spontaneously released dopamine, ultimate behavioral consequences of slow ongoing dopamine activity are less understood due to technical hurdles in achieving non-invasive and precise circuit modulation. In addition, the high-level interconnectivity of the dopaminergic network makes simultaneous manipulation of multiple cells difficult and thus precludes many studies from demonstrating causal relationship.

The brain of the fruit fly *Drosophila melanogaster* provides useful study cases in this respect. Recent studies clearly go beyond correlating physiological DAN activities and animal states, and have succeeded in examining the effect of spontaneous network activities on behaviors. These include specific DAN types that regulate sleep/wake balance, memory processing, feeding motivation or sexual drive (Berry et al., 2012, 2015; Plaçais et al., 2012; Plaçais and Preat, 2013; Cohn et al., 2015; Musso et al., 2015; Yamagata et al., 2016). Thus, this review mainly focuses on recent achievements mainly in flies, and we discuss potential roles of spontaneous DAN activity and its significance in the regulation of a variety of behaviors.

## Sleep and locomotion

Historical pharmacological studies proved that dopamine determines the arousal level in many animals. For example, methylphenidate and amphetamine, which induce dopamine release, promote arousal in humans and have been used for the treatment of narcolepsy since the 1930s (Billiard, 2008). Consistently, mutant mice with disrupted dopamine transporter (DAT) function, which has a critical role in the reuptake of dopamine, show reduced non-rapid eye movement (non-REM) sleep and increased wakefulness (Wisor et al., 2001). Furthermore, the causal nature of DAN activity in regulating behavioral arousal is gradually being revealed by recent optogenetic studies (Taylor et al., 2016; Oishi et al., 2017).

Results of physiological studies in rodents suggest that spontaneously released dopamine underlies the regulation of the wake-sleep balance. Prominent burst firing of DANs in the ventral tegmental area (VTA) was observed when animals are in REM sleep (Dahan et al., 2007). Activity of dopamine neurons in the ventral periaqueductal gray matter, but not in the VTA or the substantia nigra, is enhanced during wakefulness (Lu et al., 2006). These studies imply that the modulation of arousal is region-specific, but precise circuit level understanding remains to be revealed.

Sleep in *Drosophila* is defined by prolonged immobility and shows many common features with sleep in humans. Flies subjected to 12 h: 12 h light/dark cycles exhibit behavioral quiescence in >90% of the dark period. Sleep in flies and mammals share many characteristics. Sleeping flies show an increased threshold for sensory stimuli, and sleep deprivation by mechanical stress causes a “rebound” effect. (Hendricks et al., 2000; Shaw et al., 2000). Notably, some somnolytic drugs known to function through the human dopamine system affect *Drosophila* sleep (McClung and Hirsh, 1998; Bainton et al., 2000; Li et al., 2000; Andretic et al., 2005; Lebestky et al., 2009; Nall et al., 2016). Consistently, a fly strain that was isolated for its short-sleep phenotype was found to have a mutation in DAT, highlighting the importance of dopamine in wake/sleep regulation (Kume et al., 2005).

Until recently, the circuit mechanisms by which dopamine regulates arousal levels have been unknown. Spontaneous activity of specific types of DANs correlates well with the locomotive state of an animal and has a significant role in the regulation of the wake/sleep state (Berry et al., 2015; Cohn et al., 2015). The wake-promoting DANs project their axon terminals to two major neural structures: the dorsal fan-shaped body (dFB) and the mushroom body (MB). Below, we describe recent findings in these neural circuits.

### The dorsal fan-shaped body circuit

Dopamine released on the dorsal fan-shaped body (dFB) has a central role in the regulation of the sleep-wake balance. A single pair of DANs projecting to the dFB promotes wakefulness, and dopamine receptors are necessary in the dFB neurons to process the waking signal (Liu et al., 2012; Pimentel et al., 2016). Additional physiological experiments revealed that the spontaneous activity of DANs is increased during wakefulness (Liu et al., 2012). In contrast, the downstream dFB neurons are considered sleep-promoting (Donlea et al., 2011, 2014; Ueno et al., 2012), and are inhibited upon dopamine input (Pimentel et al., 2016). Interestingly, the membrane properties of dFB neurons reflect the animal's sleep need: sleep deprivation by continuous mechanical stress lowers the threshold to spike generation and thus increases sleep-inducing dFB neuron excitability (Donlea et al., 2014). This change is controlled by dopamine: sustained artificial activation of the DANs or sustained dopamine application for several minutes shift the states of these neurons from electrically excitable to quiescence (Pimentel et al., 2016). Therefore, spontaneous activity of these DANs regulates present and future sleep/wake balance, depending on the animal's sleep need.

### The mushroom body circuit

Besides the importance of the dFB circuit, the MB also has a critical role in the regulation of sleep (Joiner et al., 2006; Pitman et al., 2006). The MB is primarily composed of ~2,000 Kenyon cells (KCs) (Aso et al., 2009), which are presynaptic to ~20 types of mushroom body output neurons (MBONs) (Aso et al., 2014a). There are ~20 types of DANs that innervate the MB, each of which projects to a confined region of the MB, thereby controlling specific segments of the MB neurons and MBONs (Aso et al., 2014a; Hige et al., 2015). These DANs originate mainly from two clusters called PAM and PPL1 (Mao and Davis, 2009).

**Figure 1:**
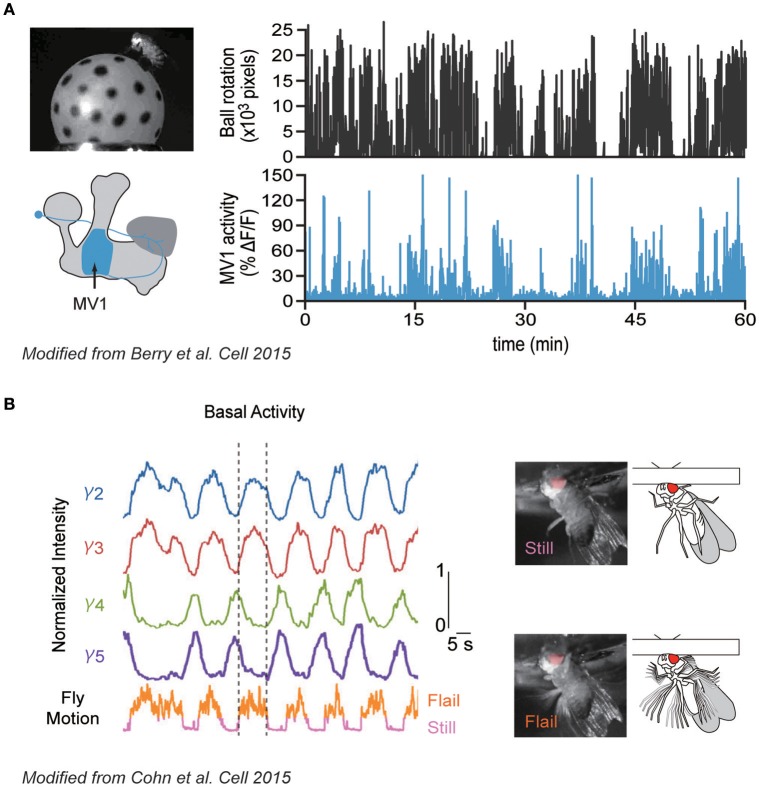
Correlative spontaneous DAN activity with the locomotive state. **(A)** Correlation between locomotive state and DAN activity. Left: Walking activity of a fly was monitored by observing the rotation of the ball during the calcium imaging of defined DAN types (MV1). Right: Walking activity of a fly (top) and calcium responses of MV1 (bottom). MV1 shows strong activity during walking bout. **(B)** Correlative and anti-correlative DAN activities with flailing. Left: Calcium responses of different DAN types innervating different MB compartments (γ2-γ5, top), and locomotive activity of a head-fixed fly (bottom). Dashed lines delineate the start and cessation of a single flailing bout. Right: Two activity states of a fly during imaging (still and flail). Modified from Berry et al. (2015) and Cohn et al. (2015) with a permission.

Berry et al. (2015) demonstrated how specific DANs, which innervate the MB-γ2α'1 compartment and are also called MB-MV1 (just MV1 hereafter; also known as PPL1-γ2α'1, show spontaneous activity that correlates with the locomotive state of the animal; namely, the calcium activity was upregulated during the walking bout (Figure 1A). Similarly, Cohn et al. (2015) found that another DAN type innervating the γ3 compartment, in addition to MV1, becomes also active during a walking bout (Figure 1B). Interestingly, spontaneous activity of DAN types innervating adjacent MB compartments (γ4 and γ5) was conversely shown to be suppressed during walking. Importantly, these DANs also respond to external stimuli such as sugar reward or electric shock in a cell-type specific manner. Therefore, both the internal locomotive state and external stimuli are integrated by the same DANs, each of which modifies a specific subdomain in the MB.

A series of experiments performed by Sitaraman et al. gives a hint of how the wake-promoting DANs exert their function (Aso et al., 2014b; Sitaraman et al., 2015a,b). Artificial activation of specific MBON types (MBON-γ5β'2, -β'2mp, and -γ4>γ1γ2), which have cell-type specific projection patterns, promotes arousal (Aso et al., 2014b; Sitaraman et al., 2015a). Interestingly, these wake-promoting MBON types receive inputs from and are activated by wake-promoting DANs (Sitaraman et al., 2015b). Note that another MBON type (MBON-γ2α'1) in contrast promotes sleep (Aso et al., 2014b; Sitaraman et al., 2015a), although the wake-promoting MV1 project its terminals to the compartment. How MV1 wakes animals has to be answered by future studies. Altogether, DANs seem to control sleep and locomotion by integrating internal states and external stimuli.

## Learning and memory

### Motivation and memory-guided behavior

It is widely acknowledged that tonic DAN activity is also involved in motivation. Microdialysis studies have demonstrated that dopamine release in slow temporal scales (tens of minutes) strongly correlates with the behavioral activity of rats (Freed and Yamamoto, 1985). Minute-by-minute dopamine levels in the nucleus accumbens correlate with an amount of reward in time and motivational vigor (Hamid et al., 2016). Sustained and ramping dopamine signaling occurs in mice moving toward predictable reward in tasks involving self-paced behavior (Howe et al., 2013). These observations collectively suggest that spontaneous DAN activity subserves motivational control of both innate and memory-guided behaviors.

How does the spontaneous DAN activity regulate learning? Recent findings in *Drosophila* give a hint for the mechanism. In flies, appetitive memory trace is thought to be localized at the synapses between Kenyon cells and MBONs (Heisenberg, 2003). The retrieval of this memory is largely dependent on the hunger state of flies (Krashes et al., 2009; Gruber et al., 2013). Krashes et al. (2009) demonstrated that such a hunger regulation is controlled by the activity of a single class of DAN cell type, called MB-MP1 (just MP1 hereafter; also known as PPL1-γ1pedc>α/β). An artificial activation of the MP1 in hungry flies during memory retrieval phase blocked the expression of memory. In contrast, a transient blockade of the same neurons restored memory expression in satiated flies. A follow-up study by the same group showed that the activity of MBONs in the corresponding MB compartment (MBON-γ1pedc>α/β) gates the expression of appetitive memory (Perisse et al., 2016). Therefore the γ1 compartment may have a central role in controlling the memory-based behavior, reflecting the huger motivation. Whether the DAN activity shapes the activity of pre- or post-synapses of the local circuitry between KC and MBON remains to be clarified in future, as both KCs and MBON can be targeted by DANs in the local circuitry of the MB lobe (Takemura et al., 2017).

### Acquisition

Besides stimulus-induced burst firing, accumulating physiological evidence revealed that spontaneous DAN activity is suppressed by the presentation of reinforcing stimuli (Brischoux et al., 2009; Matsumoto and Hikosaka, 2009; Fiorillo, 2013). This suppression can trigger memory formation: in mice, it has recently been demonstrated that repeated optogenetic silencing of spontaneous VTA DAN activity can induce place aversion (Danjo et al., 2014). Also in rats, brief suppression of spontaneous DAN activity in the VTA can substitute for negative prediction error (Chang et al., 2016), indicative of an importance of spontaneous dopamine release in reinforcement signaling.

One of the major input sources of inhibitory regulation of VTA DANs is afferents from the rostromedial tegmental nucleus (RMTg) (Jhou et al., 2009; Bourdy and Barrot, 2012; Tan et al., 2012; van Zessen et al., 2012). These GABAergic RMTg neurons receive excitatory inputs from structures implicated in aversive processing (Matsumoto and Hikosaka, 2007; Hong et al., 2011). These anatomical and functional studies in mammals provide a basic explanation for how spontaneous DAN activity can be suppressed (Danjo et al., 2014; Chang et al., 2016). However, the reinforcing property of changes in spontaneous activity of defined DANs is largely unclear.

**Figure 2:**
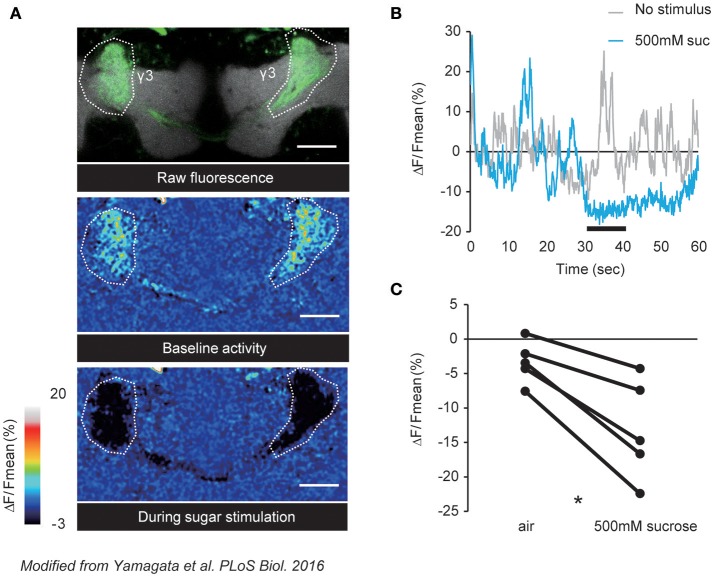
Sucrose reward suppresses the spontaneous activity of PAM-γ3. **(A)** Representative images of raw fluorescence of GCaMP expressed in the PAM-γ3 DANs (top), baseline activity (middle), and calcium responses to sucrose stimulation (bottom). **(B)** Time course of the fluorescence signal. The black bar represents the sucrose presentation. Spontaneous DAN activity is suppressed by the sucrose presentation. **(C)** Average calcium responses to sucrose presentation. Sucrose intake significantly reduces the activity level of PAM-γ3. Modified from Yamagata et al. (2016).

In *Drosophila*, distinct DANs consisting of identified cell types mediate positive or negative valences (Aso et al., 2012; Lin et al., 2014; Huetteroth et al., 2015; Yamagata et al., 2015; Aso and Rubin, 2016). It has recently been demonstrated that these valence-encoding DANs are spontaneously active (Berry et al., 2015; Cohn et al., 2015), and are dynamically tuned by external stimuli as well as the behavioral state of an animal (Figure 1). In accordance, PAM-γ3, a class of DANs projecting to the third segment of the MB γ lobe, was shown to have fluctuating baseline activity that is suppressed upon sugar ingestion (Figure 2; Yamagata et al., 2016). Interestingly, this ingestion-induced suppression of PAM-γ3 activity lasted even after the presentation of sugar reward (Figure 2B). Furthermore, transient thermogenetic and optogenetic inactivation of the PAM-γ3 was sufficient to induce appetitive memory while activation induced aversive memory (Yamagata et al., 2016). Thus, these results suggested that the spontaneous activity of PAM-γ3 represents the feeding states and that feeding drives associative memories by changing PAM-γ3 activity states regardless of increase or decrease.

Suppression of PAM-γ3 was mediated by a satiety-signaling neuropeptide, Allatostatin A (AstA) (Hergarden et al., 2012; Hentze et al., 2015; Chen et al., 2016), which is known to be a potent inhibitory neuromodulator (Birgül et al., 1999). AstA expressing neurons innervate dendritic region of the PAM-γ3, which express the AstA cognate receptor DAR-1 (Lenz et al., 2000; Yamagata et al., 2016). In contrast to aversive memory formation by PAM-γ3, activation of AstA neurons induced appetitive memory, suggesting that AstA negatively regulates PAM-γ3 spontaneous activity (Yamagata et al., 2016). Consistent with this hypothesis, down-regulation of *DAR-1* expression in PAM-γ3 diminished the feeding-induced suppression of the activity. Altogether, simultaneous recording and genetic manipulation of a DAN population in the fly brain revealed the network dynamics that determines valence (Cohn et al., 2015; Yamagata et al., 2016).

### Memory consolidation

Psychostimulants that augment dopamine signaling are known to facilitate memory consolidation. For example, avoidance learning in rats is enhanced by post-training administration of amphetamine, which increases dopamine signaling (McGaugh and Roozendaal, 2009). Post-training cocaine exposure similarly enhances consolidation of spatial memory in mice (Iniguez et al., 2012). In addition, intrahippocampal application of the D1R agonist at definite post-learning time points converts a rapidly decaying fear LTM into a persistent one (Rossato et al., 2009), suggesting a critical role for dopamine signaling in memory consolidation.

In *Drosophila*, a functional linkage between spontaneous activity of identified DANs and memory consolidation has been demonstrated. Plaçais et al. (2012) found that two pairs of DANs, MP1 and MV1, exhibit slow Ca^2+^ oscillations (~0.1 Hz) without external stimuli. In flies, repetitive training of paired presentations of odor cues and electric shocks with intervals (spaced training) is commonly used for the induction of aversive LTM (Tully et al., 1994). The authors found the regularity of slow Ca^2+^ oscillations to be enhanced after spaced training. Strikingly, suppression of the synaptic transmission of MP1 and MV1 after training diminished the formation of LTM, suggestive of a critical role of the spontaneous activity of specific DANs in memory consolidation. Since spontaneous activity of MP1 is also required for the consolidation of appetitive LTM (Musso et al., 2015), a general role may be imposed for the neural class in consolidating a labile memory into a stable, long-lasting one.

**Figure 3:**
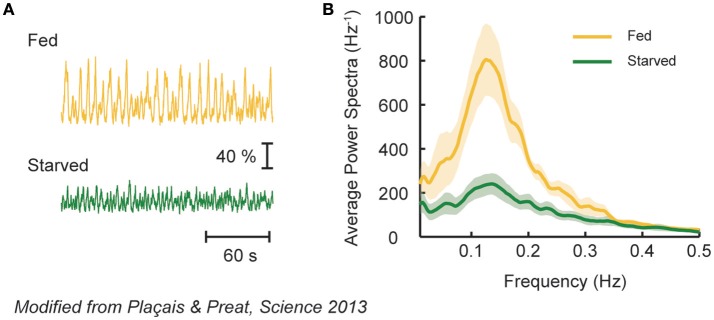
MV1 and MP1 DAN activity represents nutritive state of a fly. **(A)** Spontaneous calcium fluctuation of MV1 and MP1 DANs in fed or starved flies after spaced conditioning using electric shocks and odors. Strong calcium oscillation is observed in fed (yellow), but not in starved (green), flies. **(B)** Average power spectra of the spontaneous DAN activity in fed or starved flies. Fed flies exhibit a characteristic peak, revealing an oscillatory behavior that is absent in starved flies. Modified from Plaçais and Preat (2013) with a permission.

Spontaneous activity of MP1 and MV1 reflects the nutrient condition of an animal. In flies, the formation of aversive LTM depends on the animal's post-learning nutrient state (Hirano et al., 2013; Plaçais and Preat, 2013). This is because LTM formation is energetically costly and thus its induction is inhibited upon energy shortage (Plaçais and Preat, 2013). In accordance, the slow oscillation of MP1 and MV1 occurs only in fed flies after spaced training (Figure 3), which fits with the idea that MP1 mediates hunger motivation (see memory retrieval section). Intriguingly, driving MP1 and MV1 activity in starved flies after learning could still induce aversive LTM at the price of survival duration upon starvation, highlighting an obvious trade-off between survival and LTM formation (Mery and Kawecki, 2005; Plaçais and Preat, 2013). Thus, spontaneous activity of these DANs after memory acquisition can act as a homeostatic feedback mechanism to regulate energy state and memory consolidation.

Spontaneous activity of MP1 and MV1 has been implicated also in forgetting of short-lasting aversive memory in flies (Berry et al., 2012, 2015). Artificial activation of those DAN classes after a single training period promoted memory loss (Berry et al., 2012). Conversely, the blockade of these DANs increased the persistence of labile memory. Similar function is also imposed by a neural class belonging to the PAM cluster DANs, called PAM-β'1. Thermal activation of PAM-β'1 after learning promoted aversive memory loss (Shuai et al., 2015). Note that the identical DANs, MP1 and MV1, on one hand consolidate long-term aversive memory (Plaçais et al., 2012), while on the other hand promote forgetting short-lasting memories. Plaçais et al. (2012) showed that the memory component affected by post-training dopamine input is anesthesia-resistant memory. Intriguingly, spontaneous activity of MV1 also reflects the wake/sleep state of flies (see above Berry et al., 2015) while sleep prevents memory forgetting by blocking spontaneous activity of MV1. Given such a tight connection between sleep and mnemonic processes (Dissel et al., 2015; Haynes et al., 2015), it is plausible that the spontaneous activity of MV1 acts as a hub to link internal sleep need and memory maintenance processes.

Also in mammals, accumulating physiological evidence points to the importance of spontaneous firing of DANs in memory consolidation. For instance, spontaneous firing of VTA DANs is increased during REM sleep (Dahan et al., 2007), and is coordinated with quiet wakefulness-associated hippocampal sharp wave-ripples (Gomperts et al., 2015), which is believed to be crucial for memory consolidation (Siegel, 2001). A functional loop between the hippocampus and the VTA dopaminergic neurons was thus suggested to be crucial in post-learning DAN activity (Lisman and Grace, 2005; Gruber et al., 2016), although specific neural circuits still remain to be elucidated.

In *Drosophila*, a comprehensive anatomical study identified many feedback connections from the MB to DANs through MBONs (Aso et al., 2014a). Thus, reinforcing DANs projecting to the MB may provide an optimal study case to test the importance of the feedback regulation for memory consolidation. We previously found that a single DAN type innervating the α1 compartment (PAM-α1) has a critical role in signaling reward for appetitive LTM (Yamagata et al., 2015). Interestingly, PAM-α1 undergoes the direct recurrent regulation by MBON-α1, which has dendrites in the α1 compartment of the MB (Aso et al., 2014a; Ichinose et al., 2015). Indeed, transient blockade of neuronal components participating in this α1 feedback circuit during conditioning and early memory consolidation phase impaired appetitive LTM (Ichinose et al., 2015). This demonstrated the necessity of this recurrent circuit for LTM formation and consolidation, and suggests coordinated reverberating activity triggered by associative training (Ichinose et al., 2015). Therefore, further studies on direct measurement of post-training spontaneous activity would provide mechanistic insights to behavioral requirements of this circuit. Similar to the feedback loop between PAM-α1 and MBON-α1 for the formation and consolidation of appetitive memory, MP1 and MV1 mediate punitive reinforcement signals and are required for the consolidation of aversive memory (Claridge-Chang et al., 2009; Aso et al., 2012; Plaçais et al., 2012; Aso and Rubin, 2016). Therefore, the functional analysis of analogous recurrent circuits in aversive memory would be informative in examining the importance of this motif of dopaminergic circuits in memory formation/consolidation.

## Feeding

Dopamine is heavily involved in controlling feeding behaviors. For instance, dopamine deficient mice exhibit hypophagia, which is restored by the administration of L-DOPA (Zhou and Palmiter, 1995) or genetic rescue of dopamine production by the overexpression of tyrosine hydroxylase (Szczypka et al., 2001). The excitatory orexin inputs from the lateral hypothalamus, a neuroanatomical substrate critical for feeding (Stuber and Wise, 2016), regulate activity of VTA DANs (Aston-Jones et al., 2010). Although a causal link between spontaneous activity of VTA DANs and feeding control has been behaviorally demonstrated (van Zessen et al., 2012), mechanisms by which this activity is translated into feeding behavior are still largely unknown.

In *Drosophila*, activity of a specific class of DANs, called TH-VUM, innervating the suboesophageal ganglion (SEG), a gustatory center in the insect brain, is reported to control taste sensitivity to sucrose (Marella et al., 2012). Suppression of TH-VUM activity reduced the sensitivity while activation increased it. Strikingly, TH-VUM exhibits spontaneous activity that is upregulated upon starvation, thereby increasing responsiveness of an animal to sugar. Inagaki et al. (2012) demonstrated that starvation-induced dopamine release alters the sensitivity of sugar-sensing gustatory neurons (GRNs). On the other hand, it has been shown that spontaneous activity of a class of neurons releasing octopamine, which is an invertebrate counterpart of noradrenaline (Roeder, 1999), in the SEG, called OA-VL (Busch et al., 2009), confers bitter taste sensitivity to flies (LeDue et al., 2016). In contrast to TH-VUM, the activity of OA-VL potentiates bitter-sensing GRNs and is downregulated by starvation. In this way, starvation modulates basal dopamine and octopamine levels to control sensitivity to sweet and bitter compounds, respectively. Antagonizing activity of OA-VL and TH-VUM may thus coordinate to set a threshold for the acceptance of foods by flies.

Flies sense amino acids in food. Spontaneous activity of DANs in protocerebral posterior medial 2 (PPM2) cluster encode protein hunger (Liu et al., 2017). The activity of these neurons is upregulated after protein deprivation, and is necessary and sufficient for protein preference. Interestingly, these neurons change not only the spontaneous firing rate but also its morphology, resulting in increased number of connections with the downstream target upon amino acid deprivation. In larvae, brain DANs spanning three clusters (DM1, DM2 and DL1) also detect amino acid imbalance to reject essential amino acid-deficient diet (Bjordal et al., 2014), though this occurs based on stimulus induced DAN activity. Taken together, feeding motivation of two of the important nutrient factors, sugar and protein, is separately regulated by different DANs that monitor the need of the animal.

## Sex drive

Drugs targeting the dopamine system are known to have side effects on human sexual behavior. Spontaneous ejaculations have been reported as a side effect in patients taking Aripiprazole, which is a partial agonist of the D2 receptor (EGILmez et al., 2016). Hypersexuality and excessive masturbation in children and spontaneous erections in adults have been reported by patients taking methylphenidate (also known as Ritalin) (Bilgic et al., 2007), which primarily acts as a norepinephrine-dopamine reuptake inhibitor. Although these observations suggest a tight relationship existing between dopamine and sexual behavior (Melis and Argiolas, 1995), a functional link between spontaneous DAN activity and sexual drive has yet to be clarified in mammals.

In *Drosophila*, dopamine levels modulate both the mating drive of males as well as sexual receptivity of females: dopamine-deficient males and females respectively court and accept males less, which can be restored by L-DOPA administration (Neckameyer, 1998; Liu et al., 2008). Male flies administered methamphetamine show extremely high courtship activity, yet the latency to copulation is increased (Andretic et al., 2005). Mating drive of males is tightly regulated by their reproductive state: repeated mating progressively reduces his sexual vigor (Zhang et al., 2016). Importantly, spontaneous Ca^2+^ activity in a small subset of DANs that include aSP4 neurons in the PAL cluster are cumulatively decreased by repeated mating (Zhang et al., 2016), indicating that reproductive state is represented by the level of spontaneous activity in them. Another report suggests dopamine production in the yet another class of DANs, called PPL2ab neurons, is critical to maintain courtship activity in aged males (Kuo et al., 2015). Thus, aSP4 and PPL2ab DANs can act cooperatively for the control of sexual vigor in male flies. The target of such a motivational signal can be a group of ~20 Fruitless-expressing neurons per hemisphere called P1 (Zhang et al., 2016). P1 is a male-specific neuronal cluster that has been identified as a putative trigger center for male-type courtship behavior (Kohatsu et al., 2011; Yamamoto and Koganezawa, 2013). In accordance, protocerebral innervations of P1 and aSP4 overlap and form putative synapses (Zhang et al., 2016). Strikingly, knocking down a subtype of dopamine receptor in the P1 significantly attenuates male's courtship behaviors. It is thus conceivable that spontaneous DAN activity modulates the excitability of P1 to control sexual vigor depending on motivational state. Interestingly, this regulatory mechanism of P1 is a reminiscent of sleep/wake control in dFB, activity of which is regulated by spontaneously active DANs that reflect sleep need. Therefore, one of the major functions of spontaneous DAN activity is to represent distinct motivational states and to shape corresponding behavior by modulating the activity of key behavior-executing neurons, such as P1 or dFB.

## Detection of spontaneous DAN activity

Spontaneous activity of DANs has to be interpreted by receiving neurons through receptors. Dopamine receptors can be grouped into five classes of the guanine nucleotide-binding protein-coupled receptors (GPCRs): D1- to D5-type receptors (D1R–D5R). It is commonly accepted that D1R and D5R mainly recruit the Gα_s_ to stimulate cAMP production by adenylyl cyclase, and D2R, D3R, and D4R the Gα_i/o_ to inhibit (Beaulieu and Gainetdinov, 2011). In mammals, the inhibitory receptors show higher affinity to dopamine than the excitatory ones, and thus are suggested to play the main role in detecting the slow, tonic DAN activity (Grace et al., 2007). These inhibitory receptors are reported to be involved in the detection of wake-promoting dopamine release (Qu et al., 2010) and spontaneous activity of the value coding midbrain DANs (Bromberg-Martin et al., 2010). However, the generality and its intracellular signaling events are largely unknown.

In *Drosophila*, four dopamine receptors, DopR1 (also known as dDA1, DUMB), Dop2R, DopR2 (also known as DAMB, DopR99B) and DopEcR exist in the genome (Adams et al., 2000). Sequence homology with mammalian dopamine receptors suggests that DopR1 and Dop2R are D1- and D2- like, respectively, and the other two are invertebrate-specific (Mustard et al., 2005). Measurement of DopR2 in *Xenopus* oocyte suggested it to be excitatory (Reale et al., 1997), but recent studies suggested that it can be variable among cell types (see below). DopEcR increases cAMP upon binding to dopamine, and binds to insect steroid hormone ecdysone in addition (Srivastava et al., 2005). Affinity of these four receptors to dopamine has been respectively measured *in vitro* but with different cell lines, and never been directly compared. It is thus important to measure the threshold of these receptors for correct interpretation of functional results.

Nonetheless, accumulating behavioral and physiological evidence suggests the critical role of DopR2 in the detection of spontaneous activity of DANs. DopR2 was shown to be critical in regulation of sleep in the dFB (Pimentel et al., 2016), memory maintenance (Berry et al., 2012; Musso et al., 2015; Plaçais et al., 2017), and sex drive (Zhang et al., 2016). We will review these cases one by one.

Exquisite *in-vivo* electrophysiology experiments demonstrated that DopR2 in the dFB neurons mediates the wake-promoting dopamine signaling (Pimentel et al., 2016). This study further provided unexpected evidence that DopR2 in the dFB neurons employs Gα_o_ and thereby hyperpolarizes the membrane potential through modulating specific K^+^ channels (Pimentel et al., 2016). These results together with biochemistry experiments *in vitro* (Han et al., 1996) suggest that the nature of DopR2—excitatory or inhibitory—can be variable among cell types and imply recruitment of different Gα proteins through forming heteromeric receptor complexes.

DopR2 in the MB is also responsible for detecting the spontaneous activity of MV1 or MP1 DANs during memory maintenance (Berry et al., 2012; Musso et al., 2015; Plaçais et al., 2017). It is critical to mediate the forgetting signal of aversive short-term memory (Berry et al., 2012). It detects the nutritive value of sugar reward in appetitive conditioning to consolidate memory (Musso et al., 2015). Furthermore, it triggers energy influx to the MB that is critical for aversive LTM formation after spaced conditioning (see also above) (Plaçais et al., 2017). Strikingly, this receptor is responsible for subcellular modulation of Kenyon cell outputs in the MB lobes (Cohn et al., 2015). Not only in the MB, but also in the lateral accessary lobe DopR2 mediates increased protein feeding after protein deprivation (Liu et al., 2017). Sex drive is also regulated by DopR2 expressed in P1 neurons, which trigger male courtship behaviors (Kimura et al., 2008; Zhang et al., 2016). These studies collectively highlight a wide range of functions controlled by this receptor through detection of ongoing activity of DANs.

Two of the recent studies in *Drosophila* addressed intracellular signaling molecules that mediate the effect of spontaneous DAN activity (Cervantes-Sandoval et al., 2016; Pimentel et al., 2016). Pimentel et al. (2016) showed that dopamine/DopR2 signaling switches sleep-promoting dFB neurons from the state of excitability to one of quiescence by mobilizing potassium channels to the plasma membrane. This switch is mediated by heterotrimeric G proteins of the Gα_o_ family (Thambi et al., 1989; Pimentel et al., 2016), which deviates from the measurement in the *Xenopus* oocyte system (Reale et al., 1997). Since individual mammalian GPCRs have been demonstrated to engage multiple G proteins with varying efficacy and kinetics in a cell-specific manner, DopR2 might function as a D2-like receptor under some conditions. Cervantes-Sandoval et al. (2016) described another example of intracellular signaling events in the context of memory forgetting. Upon binding to dopamine, DopR2 activates a small GTPase, Rac1, which had been identified by another group to induce forgetting of memory (Shuai et al., 2010). This activation is mediated by a scaffold protein, Scribbled, which also can activate Pak3 and cofilin, which are key proteins in regulating actin dynamics. Altogether, Rac1, Pak3 and cofilin may thus produce necessary cytoskeletal modifications that underlie neural remodeling and consequential forgetting.

## Conclusion

**Figure 4:**
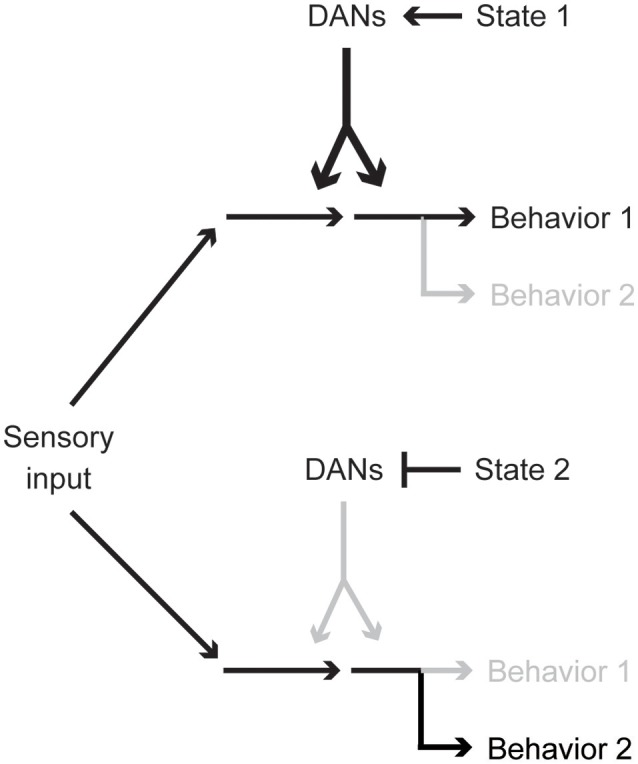
Activity pattern of a single DAN population biases behavioral choice. Particular behavioral responses are preferred when certain combinations of DAN types are spontaneously active. DAN activity reflects the physiological and psychological state of an animal and optimizes behaviors.

In this article, we have reviewed diverse functions of spontaneous activity of DANs, paying special attention to recent *Drosophila* studies. Importantly, different internal animal states, e.g., hunger, sleep need, or sexual drive, are represented by different yet partially overlapping DAN cell types. This combinatorial state coding is reminiscent of various reinforcement signals conveyed by different combinations of DANs (Aso et al., 2012; Lin et al., 2014; Huetteroth et al., 2015; Yamagata et al., 2015; Aso and Rubin, 2016). Therefore, regardless of its spontaneous or stimulus-induced origins, activity patterns of different DAN types might together be a key determinant for state-dependent behavior and action selection (Figure 4). Investigation of circuits influencing DAN activities is thus critical for understanding the cellular basis of behavioral and physiological states.

In some cases, spontaneous DAN activity functions as an information filter to enable animals to respond differently to the same sensory input, such as food, food-associated cues or potential mating partners, depending on the physiological state (Inagaki et al., 2012; Marella et al., 2012; Kuo et al., 2015; Zhang et al., 2016). Similar function of DANs to bias information flow is observed in mammalian systems (Grace et al., 2007). In addition, the spontaneous activity of specific DAN types controls ongoing spontaneous locomotor activity depending on sleep need (Donlea et al., 2011; Liu et al., 2012; Sitaraman et al., 2015b). Importantly, partially overlapping yet different combinations of DANs have an additional role in memory formation, consolidation and forgetting (Berry et al., 2012; Plaçais et al., 2012; Ichinose et al., 2015; Musso et al., 2015; Shuai et al., 2015; Yamagata et al., 2016), suggestive of the close relationship between sleep and memory formation or maintenance (Berry et al., 2015; Dissel et al., 2015). Taken together, spontaneous DAN activity patterns seem to represent both the past and present states of the animal thereby biasing behavior selection. By exploiting state-of-art genetic techniques, future studies should likely decode how animal behavior is optimized by spontaneous DAN activity.

## Author contributions

TI, HT, and NY wrote the manuscript and designed the figures.

### Conflict of interest statement

The authors declare that the research was conducted in the absence of any commercial or financial relationships that could be construed as a potential conflict of interest.
